# Role of cell wall-associated kinases in plant development and defense responses

**DOI:** 10.1007/s11033-026-12695-w

**Published:** 2026-09-01

**Authors:** Michael Dare Asemoloye, Olumayowa Mary Olowe

**Affiliations:** 1 Department of Biological & Environmental Sciences, Walter Sisulu University, Nelson Mandela Drive, Eastern Cape, Mthatha, 5117 South Africa; 2 Department of Botany, University of Ibadan, Ibadan, 200132 Oyo State Nigeria

**Keywords:** Cell wall integrity, Damage-associated molecular patterns (DAMPs), Disease resistance, Plant immunity, Wall-associated kinases (WAKs), Signaling pathways

## Abstract

Plant life is coordinated by several developmental and defense associated mechanisms and cell wall is actively important in these mechanisms. The plant cell wall also serves as an important component system that monitors plant growth and defense apart from only protecting the cell. However, resident sensors of the plant cell membrane and pattern recognition receptors (PRRs) also constitute major response mechanisms in plants while they are both as well developed for maintaining cell wall integrity (CWI). These PRRs are molecules such as ligands, peptides or glycans that are released for sensing damage collectively refers to as perceived damage associated molecular patterns (DAMPs), or signals for trigger immunity (TI). Pathogen attack frequently promotes the release of DAMPs, thereby activating TI pathways that contribute to disease resistance. The cell-wall associated kinases (WAKs) are of immense importance in this pathway. WAKs are receptor-like proteins which have their own cytoplasmic protein domain cross-linked with pectin fraction in the plant’s cell wall. They are generally known to take vital roles in cell expansion process but, it is becoming clearer that they are also involved in plant response to environmental stimuli such as pathogen attack, wounding or abiotic stresses. This review highlights the importance of understanding and identifying wall-monitoring systems involving WAKs, CWI sensors, and trigger immunity-associated PRR-DAMP interactions. This will open a new phase of how we can use these components in boosting plant immune functions and in designing better agricultural strategies such as crop breeding for disease resistance.

## Introduction

Although plants lack a mobile immune system, they possess highly sophisticated innate immune mechanisms that enable them to respond effectively to environmental challenges [1]. Plants have well-coordinated innate immune mechanisms which are based mainly on responses to environmental/external stimuli through signaling molecules and receptor proteins (RPs). The plant immune receptors for examples are grouped in two major categories based on their subcellular localization; first group are the extracellular/plasma membrane localized receptors which are majorly associated with ligands binding domain while others are grouped as the intracellular immune receptors according to recent studies on plant immune receptors [2]. Most of the extracellular/plasma membrane localized receptor proteins (LRPs) are used to perceive most external stimuli such as interference of pathogens with the apoplast. They are made up of highly conserved microbial signatures such as the pathogen associated molecular patterns (PAMPs) which can sense the pathogen’ cell wall components (like chitin) and bacterial flagella [3–5]. Another example of microbial signatures sensed by extracellular/plasma membrane LRPs is ligands that are produced by specific or group of related pathogens. Other extracellular receptors recognize pathogen-derived molecules or plant-derived signals released during pathogen invasion. while others are associated with plant derived molecules in relations to pathogen penetration [6]. In plant, the recognition of signature molecules by corresponding RPs usually triggers defense mechanism, resistance to cell death and so on (although in magnitude that differ from species to species). In turn, the ability of a pathogen to colonize a host plant depends on its virulence effectors that are secreted (usually small proteins) to weaken, disrupt or degrade some specific host defense machinery. These attributes (virulence effector) can be pathogen specific or generally common within a group of related species, they also define pathogen host range [7].

Many plant pathogens have developed advanced mechanisms with which some cytoplasmic virulence effectors (secreted extracellularly) are injected directly into the host cells to circumvent perception of the host’s extracellular/plasma membrane (LRPs). Many plants on the other hand have evolved special advanced lines of defense against the host’s defense mechanism such as the virulence effectors, this they do by forming some conserved protein families of intracellular immune receptors (IIRs). The nucleotide-binding leucine-rich repeat receptor (NLR) according to Hao et al. [8] is an example of these IIR. Many studies have also documented NLR proteins to play important roles in cereals, the NLRs usually trigger hypersensitive responses which often lead to the death of infected cells in order to maneuver the pathogen. This was first observed in dicotyledonous plant *Arabidopsis thaliana* and likened to local surface immune receptors and NLR proteins [9]. The disease resistance in plants often come as adaptive response to external stress stimuli, it can for example be quantitatively developed against a particular or many races of pathogens [10, 11]. Plants can also evolve resistance against a pathogen or disease after a period of time depending on the plant species however, this requires quantitative resistance (QR) genes which regulate resistance mechanisms in such plant [12, 13]. The WAKs are coded by very related gene clusters for examples, 30-kb locus of five gene clusters was reported in Arabidopsis, these are cytoplasmic serine threonine kinase receptor-like proteins which also contain a less conserved transmembrane domain bounded to the cell wall with series of epidermal growth factor repeats [14].

The ability of WAKs to act as pectin receptors has been proven [15], they are involved in plant response mechanisms during which plant sense pathogens exposure and produce some oligo-galacturonic acid (OG) fragments. The ability of WAKs to respond and bind different kinds of cell wall pectins affirms their roles in pathogen resistance and cell growth. Knowledge of WAKs originated from early cDNA studies that unexpectedly revealed proteins associated with the plant cell wall and plasma membrane [16, 17]. They were defined as wall associated receptors that could be extracted from plant cell wall/membrane or viewed through the use of electron micrographs however, treatment of protoplasts with protease and western blots also affirm their cytoplasmic domain with the cell membrane and their five genes are closely clustered in the chromosome. The WAKs receptor is cross linked in such a way that the extracellular air space houses the epidermal growth factor domain [18].

Although WAKs have traditionally been investigated in separate contexts such as cell expansion, pathogen resistance, and abiotic stress adaptation, growing evidence indicates that these functions are mechanistically interconnected. The common feature underlying these diverse processes is the ability of WAKs to sense alterations in the cell wall and relay such information to intracellular signaling pathways. Consequently, WAKs can be viewed as central components of the cell wall integrity (CWI) surveillance system that integrates developmental cues with defense responses [19]. To provide a more unified understanding of WAK biology, this review follows the progression from cell wall integrity sensing to WAK-mediated signaling, and finally to the developmental and disease-resistance outcomes regulated by these receptors.

## Plant cell wall integrity and disease resistance

### The plant cell walls and pathogens

Since WAKs function at the interface between the cell wall and plasma membrane, understanding their biological significance requires first examining the role of the cell wall in plant defense. The plant cell wall is not simply a static protective barrier; rather, it is a dynamic structure that continuously responds to developmental signals and environmental perturbations. Changes in cell wall composition and architecture are closely monitored by surveillance systems that activate downstream signaling pathways whenever integrity is compromised [20]. Thus, cell wall integrity forms the foundation upon which WAK-mediated growth and defense responses are built.

**Fig. 1:**
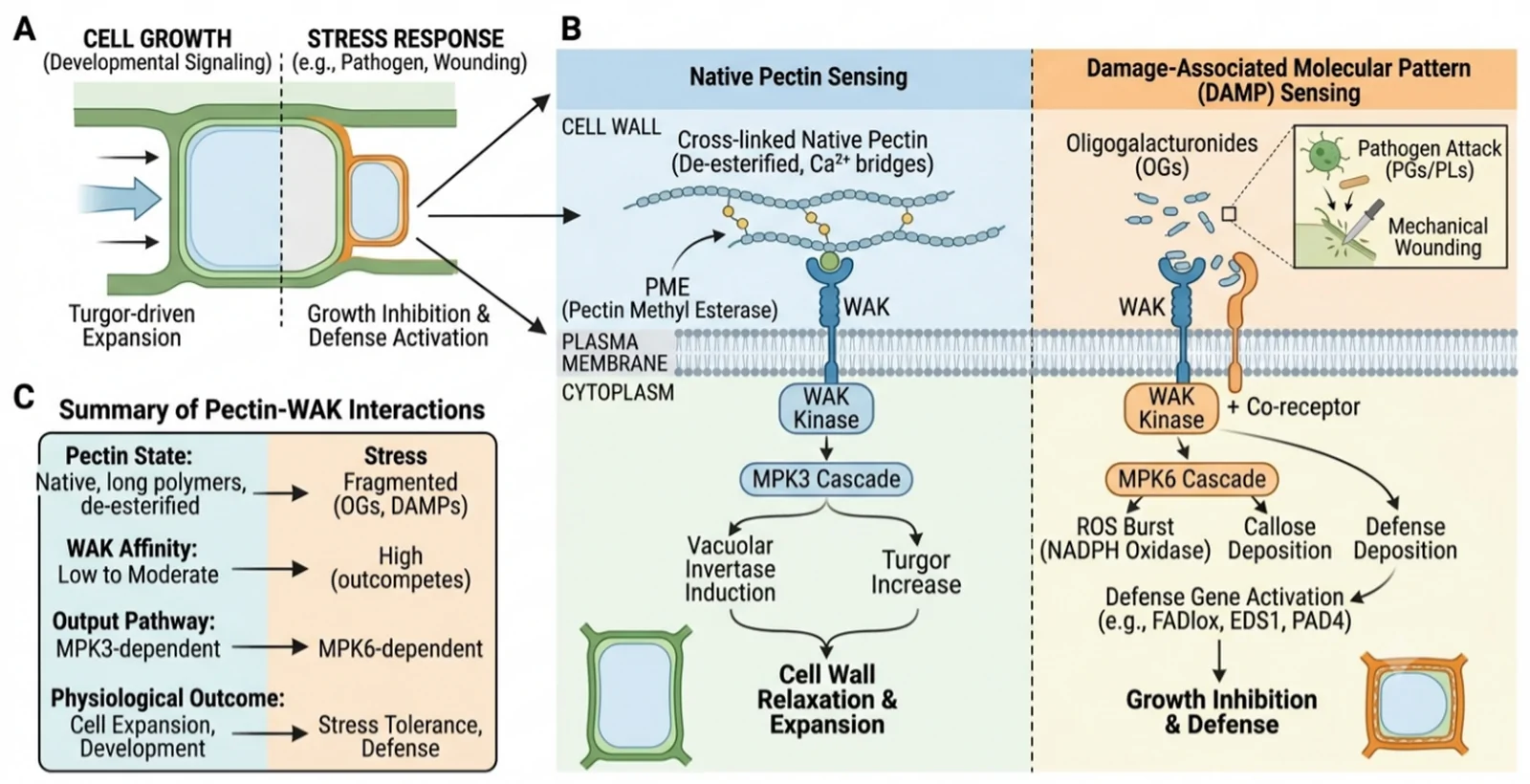
Model of pectin-WAK signaling in plant cell expansion and stress responses. **a** Physiological transition between turgor-driven cell expansion during growth and growth inhibition during stress. **b** Molecular mechanisms of pectin sensing across the plasma membrane. Under normal developmental conditions (left), long-chain native pectin de-esterified by PMEs binds WAKs to activate an MPK3 cascade, driving vacuolar invertase expression and cell wall expansion. Upon pathogen attack or mechanical injury (right), oligogalacturonide (OG) fragments act as DAMPs, binding WAKs with high affinity to trigger an MPK6 cascade, ROS production, callose deposition, and defense gene transcription. **c** Summary matrix comparing pectin structural states, WAK binding affinities, kinase pathways, and physiological outputs. DAMP damage-associated molecular pattern, MPK mitogen-activated protein kinase, OG oligogalacturonide, PME pectin methylesterase, ROS reactive oxygen species, WAK wall-associated kinase

Cosgrove [21] established the dynamism and highly controlled plant cell wall which is vital for growth and development of the plant cell. However, it is yet unclear about the mechanism behind plant cell wall’s modification and synthesis and how they are being communicated to the plant cell. Aziz et al. [22] emphasized on the plant cells surrounded by primary cell which composed of carbohydrate-based polymers such as cellulose which may harbour series of biochemical modification such as esterification (Fig. 1). Also, the cells with completion of cellular expansion and reinforced wall structure such as formation of xylem with new inner layers with secondary cell wall enriched in xylan and lignin, as well as cellulose [23].

The plant cell wall is protected from disease development through the antimicrobial means of killing potential intruders, the inhibition of enzymes that degrade cell wall, building of structural barriers and chemical remodeling at the sites of penetration [24]. Antimicrobial proteins, phytoalexins and phytoanticipins in plants are compounds with low weight which resist and restrict fungi [25]. Also, one of the major player of resistance in plant is the production of phytoalexin due to its inhibition of disease development which may also be essential for penetration resistance and mechanical strengthening of the plant cell wall. Collectively, these studies demonstrate that plant defense is not solely dependent on antimicrobial compounds but also on the ability of the cell wall to function as an active surveillance system. During pathogen attack, remodeling of cell wall components and release of wall-derived fragments generate signals that are subsequently perceived by receptors such as WAKs. Therefore, biochemical changes in the cell wall represent an important link connecting pathogen invasion with activation of downstream defense signaling pathways. Gamir et al. [26] established the importance of indole-3-carboxylic acid during the plant and pathogen interactions.

It is known that the hydrolytic enzymes that are produced by plants often bind to glucosides to form poisons against pathogens, it is although how sites of fungal attack are targeted by antimicrobial compound. Mireku [27] reported the production of fungitoxic 3-desoxy anthocyanin flavonoids in Sorghum bicolor in specific site when attacked by *Colletotrichum* species. In non-membrane bound cellular inclusions of this chemical usually attack lipid bilayers to increase cell collapse/death simply, the phytoalexin migrates through the apoplast into pathogen location or attack sites.

### Role of WAKs and pectins in cell growth plant’s response to external stimuli

The defense-related functions of the cell wall are closely linked to its developmental functions. During normal growth, cell wall remodeling is required for cell expansion, differentiation, and organ formation. Because WAKs are physically connected to pectic components of the wall, they are ideally positioned to coordinate these developmental processes. Therefore, the same receptor systems that monitor cell wall damage during stress conditions may also regulate cell expansion and developmental signaling under normal physiological conditions.

Wall associated kinases were initially identified during molecular characterization studies through the isolation of cDNAs that revealed their importance to cell wall, and they were regarded in different explanations as wall associated receptors. Also, these proteins can only be extracted from cell membranes or walls through detergent boiling which is then seen cross linked with insoluble material and seen under electron microscopy powered with specific WAK-kinase antiserum. The protease treated protoplasts and western blot experiments have shown that kinase domain is located in the cytoplasm but cross-linked membrane towards the exodermally placed epidermal growth factor (EGF) which is located at the extracellular space [17, 28]. It is also good to note here that many models have been built to further prove that regulation of cell expansion by WAKs is actively via the mechanism of sugar and turgor control. This model was supported by Yao et al. [29] that the *wak2-1* phenotype is an expression of sucrose phosphate synthase an enzyme that can alter sugar sinks however, how WAKs influence turgor regulated cell wall expansion is not yet fully understood.

The WAKs as plasma membrane receptors are cross linked with cell wall materials and they can be observed in an electron microscope. They are released from the cell wall materials by the pectinase and not the cellulose or other cell wall enzymes [30], although it was previously suggested that WAKs are covalently bonded with pectins [31, 32]. WAK proteins bind to pectin on denaturing gels and bonding of purified extracellular domains of *WAK1* or *WAK2* with pectin have been demonstrated in vitro [33, 34]. They have higher affinity for de-esterified pectin than non-negatively charged esterified molecules [35, 36]. Importantly, the kind of pectin that WAKs bind to in plants, it was demonstrated in an invitro experiment by Kohorn and Kohorn [16] that *WAK1* and *WAK2* bind to different kinds of pectins such as polymers of homo-galacturonans, oligogalacturonic acids (OGs) and rhamnogalacturonans I and II. These pectins all contain galacturonicacid backbone, they therefore predicted that presence of galacturonic acid is the requirement for WAKs binding to pectins.

Although these studies were conducted using different experimental systems, they collectively support a common conclusion: WAK proteins are uniquely positioned to perceive structural modifications occurring in cell wall pectins. This perception enables WAKs to act as intermediaries between extracellular wall dynamics and intracellular signaling processes, thereby explaining their involvement in both developmental regulation and defense responses. The role and biological importance of pectin fragments or OGs have been widely studied for many years and they are affirmed to directly influence plant growth, defense and response to external stimuli or stresses [37, 38]. The WAKs also play important roles in cell expansion and plant responses to pathogens and stresses, as these induce their expression [29, 32]. This induction is however passive in signaling response in plants and not necessarily indicating the role of WAK receptor itself. Collectively, these findings indicate that WAK-mediated signaling is largely dependent on the physiological state of pectin within the cell wall. Rather than acting as isolated pectin receptors, WAKs appear to function as molecular interpreters that convert information contained in pectin structure and integrity into developmental and defensive outputs. This concept provides a unifying explanation for why WAKs are involved in both growth regulation and pathogen-induced responses across diverse plant species.

The structural organization of WAK proteins provides the mechanistic basis for their diverse biological functions. Through their extracellular domains, WAKs perceive changes occurring within the cell wall matrix, whereas their intracellular kinase domains transmit these signals into downstream regulatory networks. This dual functionality positions WAKs as important signaling intermediates capable of translating alterations in cell wall status into developmental responses. Consequently, many developmental processes regulated by WAKs can be viewed as direct consequences of cell wall perception and signaling.

While WAKs perceive changes occurring at the cell wall interface, their biological effects depend on intracellular signaling events. One of the principal mechanisms through which WAK-derived signals are transmitted involves protein kinase cascades. Therefore, understanding the contribution of protein kinases to plant defense is essential for appreciating how extracellular cell wall perturbations are translated into cellular responses [39]. In this context, WAKs should not be viewed as isolated receptors but as upstream components of broader signaling networks involving MAPKs, RLKs, ROS signaling, and defense-related gene activation.

### Mechanistic basis of WAK-mediated regulation of plant development and defense responses

**Fig. 2:**
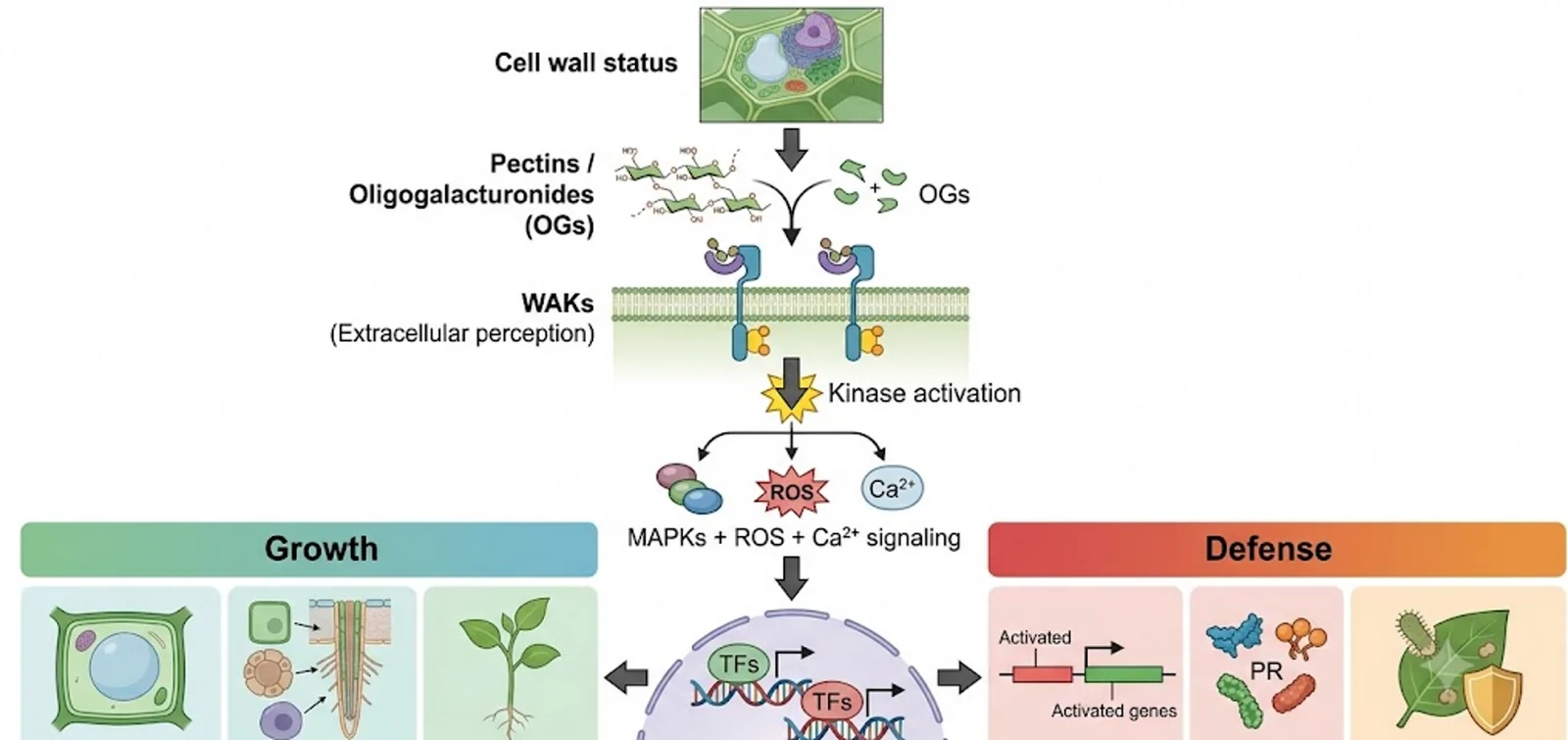
Mechanism of WAK-mediated regulation of plant development and defense responses. Each component is illustrated with relevant molecular and cellular details,; the top (Perception) shows the cell wall and the binding of pectin fragments (OGs) to WAK receptors. Middle (Signaling) details the intracellular activation cascade involving ROS, calcium, and MAPK signaling that leads to the activation of transcription factors in the nucleus. Bottom (Response) clearly separates the outcomes into a “Growth” pathway (left, showing cell expansion and plant development) and a “Defense” pathway (right, showing immune gene activation and disease resistance)

Wall-associated kinases (WAKs) function as molecular bridges linking the extracellular matrix of the plant cell wall with intracellular signaling networks. Structurally, WAKs possess an extracellular domain that binds pectic polysaccharides within the cell wall, a transmembrane domain, and a cytoplasmic serine/threonine kinase domain capable of initiating signaling cascades (Fig. 2). This unique organization allows WAKs to perceive alterations in cell wall integrity and translate them into physiological responses through intracellular signal transduction pathways [16, 28].

Key Mechanistic Steps of WAK Signaling include:

i. Cell wall remodeling or pathogen attack alters pectin structure.
ii. Pectins or oligogalacturonides bind extracellular WAK domains.
iii. WAK kinase domains become activated.
iv. MAPK and ROS signaling pathways are triggered.
v. Defense- and growth-related genes are transcriptionally regulated.
vi. Developmental adaptation or immune response is produced.

Under normal developmental conditions, pectin remodeling accompanies cell elongation, differentiation, and tissue expansion. WAKs interact preferentially with pectin molecules, particularly negatively charged de-esterified pectins, enabling them to monitor cell wall status continuously [35, 36]. Studies in *Arabidopsis thaliana* demonstrated that *WAK2* regulates vacuolar invertase activity, sugar metabolism, and turgor-driven cell expansion. Mutations in *WAK2* impair root growth under low-sugar conditions, highlighting a direct role for WAK-mediated signaling in developmental regulation [28, 40]. These findings suggest that WAKs coordinate cell wall dynamics with metabolic processes required for plant growth.

When plants encounter pathogens, herbivory, or physical injury, cell wall degradation releases oligogalacturonides (OGs), which serve as damage-associated molecular patterns (DAMPs). WAKs recognize these pectin-derived fragments and initiate defense-related signaling pathways [16, 38]. Binding of OGs to WAK receptors activates their kinase domains, leading to phosphorylation cascades involving mitogen-activated protein kinases (MAPKs), calcium influx, and reactive oxygen species (ROS) production [36, 41, 42]. These signaling events subsequently trigger transcriptional reprogramming and activation of defense-associated genes.

Accumulating evidence indicates that WAK-mediated signaling serves as a convergence point integrating developmental and immune signaling pathways. During normal growth, perception of structural pectins promotes cell expansion and maintenance of cell wall integrity. In contrast, during stress conditions, perception of OGs and other wall-derived signals redirects cellular resources toward immune activation and repair processes [35, 38]. This functional duality enables plants to dynamically balance growth and defense according to environmental conditions.

Downstream of WAK activation, MAPK signaling pathways play crucial roles in amplifying cellular responses. Activation of MPK3 and related kinases has been reported following pectin or OG perception, leading to enhanced expression of defense genes, ROS accumulation, and pathogen resistance responses [36, 43]. Furthermore, several WAK and WAK-like proteins have been associated with enhanced resistance against fungal and bacterial pathogens in rice, wheat, maize, Arabidopsis, and rose, demonstrating that WAK-dependent signaling mechanisms are evolutionarily conserved across higher plants [16, 44–47].

Collectively, current evidence supports a mechanistic model in which WAKs act as central sensors of cell wall integrity. Changes in pectin structure generated during either developmental remodeling or pathogen-induced cell wall damage are perceived by extracellular WAK domains. This perception activates intracellular kinase signaling, which subsequently regulates MAPK cascades, ROS production, defense gene expression, carbohydrate metabolism, and cell wall biosynthesis pathways. Through these integrated mechanisms, WAKs coordinate plant growth, development, stress adaptation, and disease resistance [16, 28, 29].

### Significance of protein kinases in plant’s response to biotic stress

Proteins generally constitute major part of plant cell wall, they confer it appropriate properties/modification and generally on the cell [48]. Also, they contribute to signaling by releasing oligosaccharides which is the signal molecules or through interaction with plasma membrane receptors, resulting in a diverse array of proteins within the plant cell wall [6, 49]. Interaction with other cell components, differences in their relative abundance and physico-chemical properties (Table 1) have been observed [39]. Plant resistance to pathogen depends largely on its ability to recognize such pathogen early. The reactive oxygen signaling (ROS) is generated by plants via different processes to control various biological programs [50]. The ROS are products of aerobic metabolism in plants which are commonly produced after pathogen attack, then followed by hypersensitive response as such, they are commonly involved in disease resistance and in signaling responses of ABA phytohormone [51, 52].

The involvement of some plant protein kinases in the cross-linked between abiotic and biotic stress has been proven. Pathogenic response increase expression of different plant protection, defense genes, activate protein kinase and create some specific genetic mechanisms like the cell wall proteins crosslinked with pectins [6]. Evidence of the protein kinase involvement in the plant defense activation was reported by [41, 53, 54].Although numerous protein kinases participate in plant immunity, they are discussed here because WAK-mediated signaling ultimately converges on broader kinase networks. Consequently, understanding MAPKs, RLKs, and ROS-associated protein kinases provides insight into how signals initiated through cell wall-associated receptors are amplified and translated into biological responses.

According to Zhang and Zhang [20], the protein kinase enhances a wounding-activated kinase of about 46 kDa and this activation is induced by pathogen elicitor. The induction and development of PR1 gene expression is majorly enhanced by protein kinase in protein phosphorylation which helps in the defense signaling pathway [53]. A study was reported by Kohorn and Kohorn [16] and Kohorn [28] about the activity of MAPK found in tomato and tobacco leaves due to biotic stress of wounding. Nie et al. [64] pointed at the mounting evidence which indicates the rapid and transient protein phosphorylation triggered by fungal elicitors. It has been observed that some protein kinases such as SFR2 and PR5K receptor-like kinases of *Brassica oleracea* and Arabidopsis respectively are involved in disease resistance [65]. The PR5K receptor-like kinase have an extracellular domain homologous to a PR-5 protein which is an antimicrobial protein involved in detecting the pathogen signals also, the SFR2 which is a receptor-like kinase is likely to contribute to this signaling mechanism after wounding or bacterial infection [16]. Also, MAPKs signaling pathway maybe activated by various pathogens to induce the pathogenesis-related (PR) gene as a result of increased level of ROS in the cell. Ligterink et al. [66] established the MAPK activation where there is an induction of Pep13, stimulating PR gene during resistance towards pathogens.

The signaling activities described above ultimately influence plant phenotypes through genetically controlled defense pathways. Consequently, understanding WAK function requires moving beyond biochemical signaling events to examine the genetic architecture underlying disease resistance. Numerous resistance loci, quantitative resistance genes, and kinase-associated genes have now been linked to plant immunity, providing a genetic framework through which WAK-mediated responses can be interpreted.

**Table 1:** The role of some plant protein kinases during biotic stress

Protein Kinase	Type/Class	Plant Species	Role During Biotic Stress	Pathogen Target/Response	Reference
BIK1	RLCK-VII	*Arabidopsis thaliana*	PTI signaling via FLS2/EFR	*Pseudomonas syringae*	[55]
MPK3/MPK6	MAPK	*Arabidopsis*, *Tomato*, *Apple*	Defense gene activation, HR	Broad-spectrum defense	[56]
CDPKs (e.g., CPK3, CPK5, CPK11)	Calcium-dependent	*Arabidopsis*, *Rice*, *Tomato*	ROS production, defense signaling	Fungal and bacterial pathogens	[57]
WAK1	RLK	*Tomato*, *Arabidopsis*	Cell wall damage sensing	*P. syringae*, *Fusarium spp.*	[58]
OsRLCK176/185	RLCK	*Oryza sativa*	Chitin-triggered immunity	Fungal pathogens	[59]
OXI1	AGC kinase	*Arabidopsis*	ROS-mediated MAPK activation	Oxidative stress and pathogens	[60]
SnRK2.6	SnRK	*Arabidopsis*, *Maize*	ABA and immune signaling	Hormone-pathogen cross-talk	[61]
PEPR1/PEPR2	RLK	*Arabidopsis*	DAMP recognition and immune amplification	General pathogen defense	[55]
WTK1 (Yr15)	Tandem Kinase	*Wheat*	Resistance to stripe rust	*Puccinia striiformis*	[62]
Rpg1	Tandem Kinase	*Barley*	Durable stem rust resistance	*Puccinia graminis*	[62]
SlMAPKs	MAPK	*Solanum lycopersicum*	Signal transduction, stress adaptation	*Botrytis cinerea*, *P. syringae*	[63]
MdMAPKs	MAPK	*Malus domestica*	Defense signaling	*Erwinia amylovora*	[58]
VvMAPKs	MAPK	*Vitis vinifera*	Immune signaling	*Plasmopara viticola*	[58]
CsMAPKs	MAPK	*Cucumis sativus*	Biotic stress signaling	*Colletotrichum orbiculare*	[58]
FvMAPKs	MAPK	*Fragaria vesca*	Defense and hormone signaling	*Botrytis cinerea*	[58]

This table is showing the diversity of kinase-mediated defense mechanisms across different plant taxa. The kinases mentioned therein are involved in signal transduction, pathogen recognition, and activation of immune responses such as ROS bursts, MAPK cascades, and transcriptional reprogramming. They often function downstream of pattern recognition receptors (PRRs) or in cross-talk with hormone signaling pathways. In addition, they are central to pattern recognition, signal transduction, and immune activation, often functioning in complex cascades involving MAPKs, CDPKs, and RLCKs. They also mediate cross-talk between abiotic and biotic stress responses, making them key targets for crop improvement

## Genetics basis of disease resistance and roles played by wall associated kinases

Accumulating evidence from multiple plant systems supports a conserved role for WAK-mediated genes in integrating growth and defense responses. Although individual studies have examined different species and stress conditions, a common pattern emerges whereby WAKs regulate cell wall integrity perception, activate downstream signaling pathways, and ultimately contribute to disease resistance [20]. Selected examples from Arabidopsis, rice, maize, and other crops illustrate this conserved biological framework.

### Genetic insights into plant disease resistance

There are two types of disease resistance in plants; the qualitative and quantitative resistance. Qualitative resistance is controlled by major R (genes) which confers a total resistance to specific pathogen races which is useful for crop improvement and basic research. These genes have been reported to be cloned and virulent pathogens can rapidly be overcome by resistance mediated by these genes. The multiple genes control the quantitative resistance with minor impact and each of the gene contributing to partial resistance [10]. There is lower selection against pathogen variants by quantitative disease resistance and there is no or little advantage for those that overcome an individual quantitative resistance locus (QRL), hence it stands to be more effective compared to the resistance that is controlled by R genes [67].

While many resistance genes function independently of WAKs, their discussion is relevant because they illustrate the broader genetic architecture through which plants coordinate disease resistance. WAK-mediated pathways interact with these immune mechanisms and contribute to quantitative disease resistance by facilitating early perception and signaling events linked to cell wall integrity.

Studies have reported the significance of quantitative disease resistance in basic research and crop improvement. Marker-aided or traditional methods of plant breeding can exploit the variation in quantitative disease resistance which may result in an acceptable level of disease control [68]. Jiang et al. [69] reported the third class of R genes in which the rice *Xa21* gene is being characterized, this gene confers resistance to bacterial pathogen as it features a transmembrane protein kinase and extracellular LRRs. The fourth class of resistance gene is the arabidopsis *RPW8* protein, this has a putative coiled-coil domain (CC) and a membrane protein domain respectively [70].

Quantitative resistance loci (QRL) are biological activities that may involve the control of physical and chemical developmental processes which assist with defense signal transduction, promoting basal defense, circadian clock-associated genes, encoding enzymes to detoxify pathogen-produced phytotoxins and a set of unidentified genes and weaker alleles of R genes. Zhang et al. [71] also reported a correlation between camalexin with quantitative disease resistance based on biochemical study of Arabidopsis-Botrytis pathosystem, and contribution of camalexin sensitivity of different pathogenic specificity which indicate that QRL may be related to anti-pathogen chemicals production. Expression of some pathogenesis-related proteins responsible for resistance to biotrophic maybe as a result of salicylic acid (SA)-dependent signaling pathway activation [72]. There is an extensive interaction between JA, SA and ethylene signaling pathways [73]. It has been established that there are several signaling components in plants that regulate regulate different levels of susceptibility when mutation occurs which result to several types of alleles involved in the signaling pathways regulation speculated to be QRL [74]. It is being reported that non-classical resistance genes control quantitative disease resistance. Brown [75] established that the non-classical gene *pi34* located in a 65.3-kb, is partially resistance to rice blast which spans 10 open reading frames. Some QRL such as *pi20* gene in rice have not been associated with disease resistance however, this is not similar to the known defense-related genes which encode for mutated proline-rich proteins [71]. Mapuranga et al. [76] emphasized on the *Yr36* in adult wheat plants which confers resistance to a broad spectrum of stripe rust races under high temperature (25 –35 °C) as well encode both putative START (steroidogenic acute regulatory protein-related lipid transfer) and kinase.

## Kinase mediated genes and their roles in disease management

Kinase mediated genes are transmembrane domain receptor-like protein, they are cytoplasmic serine threonine kinase located in less conserved regions bounded with plant cell wall and as well contain different types of epidermal growth factor repeats. Their ability of genes associated controlling the WAKs responses to varieties of pectins in plants affirms their roles in growth and disease resistance [29, 42]. Although many studies affirm the importance of WAKs proteins in cell expansion and plant response to pathogen attacks, genes controlling this mechanism are still poorly understood and documented [16, 47].

Evidence from multiple plant species consistently indicates that WAK-mediated genes influence both plant growth and disease resistance through a shared mechanism involving cell wall integrity perception. Although individual studies have focused on different species and stress conditions, together they reveal a conserved role of WAK signaling in translating extracellular wall-derived signals into developmental and immune responses. In a study, dominant mutant of WAK alleles were observed to provide resistance in Arabidopsis against *Fusarium* pathogen while they were less sensitive to rice blast fungus [44, 47]. It was revealed in a study that a *WAK2* null allele hinders the expansion of root cells under low sugar and salt condition [40]. It was also affirmed in a root enzyme activity survey by Kohorn et al. [40] that there was reduced vacuolar invertase activity in *wak2-1* null mutants, this may be a reflective of the fact that *WAK2* can influence the transcriptional invertase RNA levels. Although loss of function in any of the four alleles result not in any phenotype. In Arabidopsis for example, all the five WAKs genes lie in a 30 kb cluster and by this it is difficult to create their double or triple mutants of the isoforms [28]. The *WAK1* has dominant alleles for high salicylate resistance but have no extracellular domain while antisense *WAK1* plants are usually very sensitive to salicylate according to Stephens et al. [47].

It is however not very clear in their work how WAKs are involved but they were able to establish its connection with pathogenesis, more is needed to explore roles play by OGs in regulation of WAKs for disease resistance. More roles of WAKs in pathogen response by plants was revealed in a study carried out by Kohorn [28]; Kohorn et al. [36]. They reported that dominant allele of *WAK2* cTAP was observed to cause sudden (ectopic) lesions, Reactive oxygen species (ROS), leaf curling, stunted growth and cell death. They explain that mutant cTAP alleles present in pectin binding domain, epidermal growth factor (EGF) or catalytic kinase site do not induce this phenotype do not induce this phenotype this means that for this to happen, there is need for an active receptor. In addition, Kohorn et al. [36]; Kohorn and Kohorn [16] observed that receptor of *WAK2cTAP* is responsive to pectin stimulation and can appear as a hyper-activated allele. Mitogen activated protein kinase (MAPK) is a protein kinase that is widely reported to be specific to serine and threonine amino acids in directing cellular response to different stimuli with respect to proliferation, differentiation, mitosis, survival, apoptosis and expression of genes.

Kohorn et al. [36] observed an elevated activity of MPK3 in plant cells treated with long pectin polymer while Su et al. [41] and Giovannoni et al. [77] showed that OGs can activate MPK3. Structurally, plant cell wall is formed by a complex regulated developmental process from synthesis, turnover to protein and carbohydrate interactions, with some metabolic and developmental pathways. This must have involved several alleles of cell wall biosynthesis genes of which receptor kinase is prime importance [39]. Many of receptor kinase have been identified; examples include THESEUS (THE1) [78, 79], FERONIA (FER), HERKULES (HERK), and ANXUR [19, 80, 81]. This class of receptor has been reviewed extensively elsewhere that they have been termed cell wall sensors [19, 81].

**Fig. 3:**
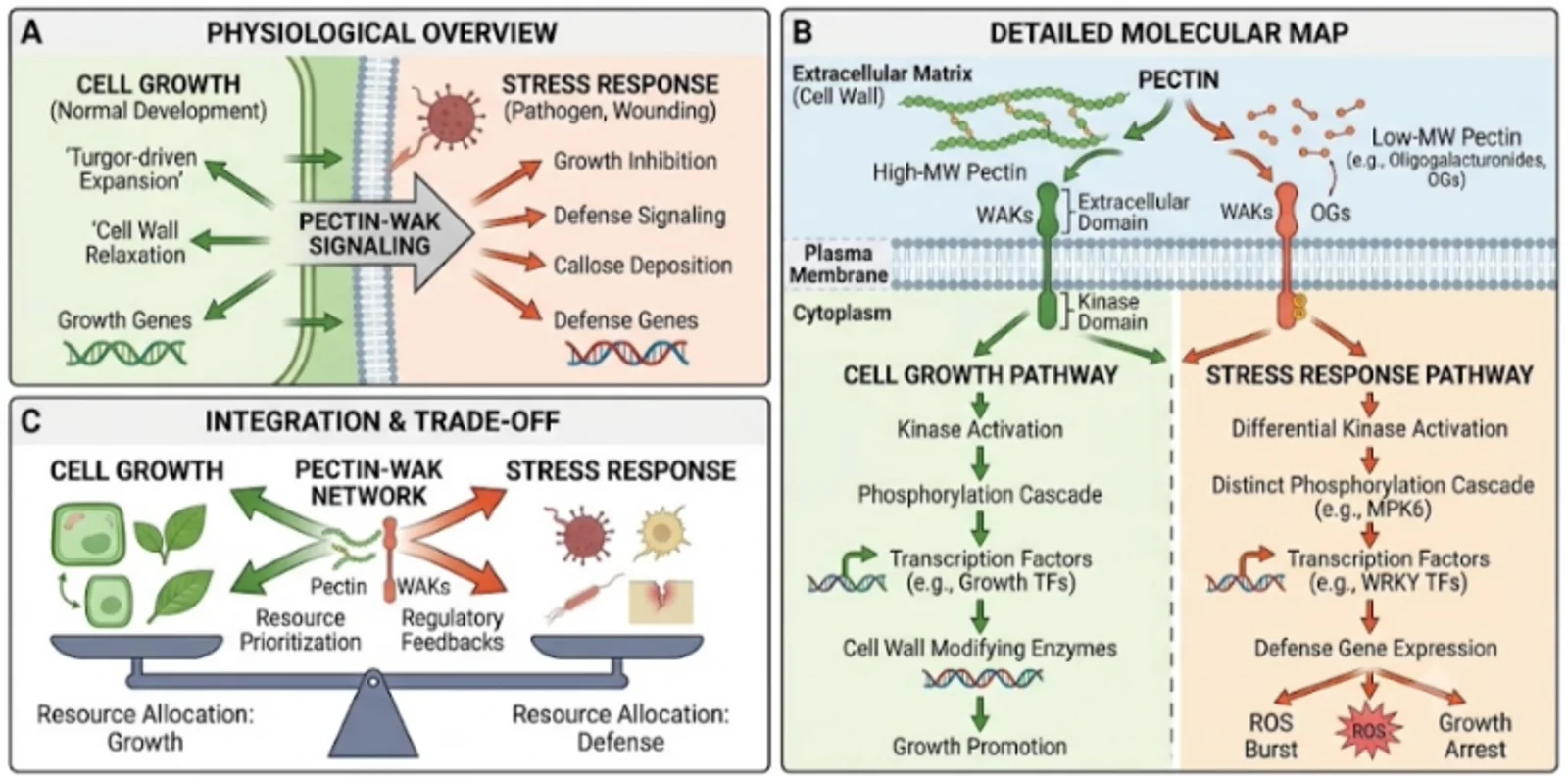
Schematic representation of the pectin-WAK signaling network regulating plant cell expansion and stress responses. (**A**) Macro-level physiological outcomes showing the dual function of wall-associated kinases (WAKs) in modulating turgor-driven cell expansion during vegetative growth versus cell growth inhibition and defense activation during pathogen attack or wounding. (**B**) Molecular mechanism of pectin perception at the plasma membrane. Under normal growth conditions (*left*), long-chain, de-esterified native pectin processed by pectin methylesterases (PMEs) interacts with WAKs to activate an MPK3 signaling cascade, leading to vacuolar invertase induction and cell wall loosening required for elongation. Upon stress (*right*), polygalacturonases and pectin lyases cleave cell wall pectin into oligogalacturonide (OG) fragments. These OGs act as damage-associated molecular patterns (DAMPs) and bind WAKs with high affinity, activating an MPK6 cascade that drives NADPH oxidase-dependent reactive oxygen species (ROS) production, callose deposition, and upregulation of defense-related genes (*EDS1*, *PAD4*, *FADlox*). (**C**) Comparative matrix summarizing key interactions between non-stressed (growth) and stressed (defense) pectin-WAK pathway components. *DAMP* damage-associated molecular pattern, *EDS1* enhanced disease susceptibility 1, *FADlox* fatty acid desaturase/lipoxygenase, *MPK* mitogen-activated protein kinase, *OG* oligogalacturonide, *PAD4* phytoalexin deficient 4, *PME* pectin methylesterase, *ROS* reactive oxygen species, *WAK* wall-associated kinase

WAKs regulate plant development and defense through a coordinated signaling mechanism that begins with perception of pectin-derived cell wall signals and culminates in activation of intracellular kinase cascades [16]. Depending on the nature of the signal perceived, WAK-mediated pathways may promote either developmental processes such as cell expansion and differentiation or immune responses involving ROS production, MAPK activation, and defense gene expression. Thus, WAKs function as central integrators linking extracellular cell wall status with intracellular biological responses. WAKs are unique as they can bind cell wall pectin and effect a WAK-dependent signaling pathway for regulating the cell expansion. They have also been shown to involved in plant response to external stimuli and pathogens. It should be noted that the regulatory action of WAKs through cell wall pectins are greatly aided by their structural requirements or the pectins which in turn also require numerous signaling pathways (Fig. 3).

The evidence presented thus far demonstrates that WAKs and related kinase genes contribute significantly to disease resistance through cell wall sensing and signaling mechanisms. These discoveries have important practical implications because they provide potential molecular targets for crop improvement programmes. Therefore, translating fundamental knowledge of kinase-mediated resistance into breeding applications represents a logical next step in the utilization of WAK-associated pathways. In addition, discoveries regarding WAK-mediated signaling have important implications beyond basic plant biology. The demonstrated roles of WAKs in disease resistance and stress adaptation have encouraged efforts to exploit kinase-associated genes as targets for crop improvement, particularly through conventional breeding, marker-assisted selection, and modern genome editing approaches.

## Exploitation of kinase genes for plant disease resistance

Many evidences have been published towards the roles of WAKs in cell growth and responses, in results reported by Yao et al. [29] and Stephens et al. [47], the antisense RNA of WAK can be induced using Dex system in conserved WAK-kinase domains leading to 50% reduction in WAK protein levels and also can result in production of smaller size unlike smaller cell number meaning that WAKs play crucial role in cell elongation. In another study, a null allele *AWAK2* under reduced salt and sugar conditions caused loss of cell expansion in plant roots while losses in four other WAK alleles did not resulted in any visible phenotype [28, 40]. There was an observation in an enzyme activity survey as reported by Kohorn [28] that the roots of *wak2-1* null mutants had a reduced invertase enzyme activity in its vacuoles, this also affects the invertase RNA levels even at the transcriptional level and this is an evidence that *WAK2* can regulate the cell expansion. It is also evident that WAKs can control turgor potential of cells via its effects on sugar concentrations, and this model suggests that *wak2-1* phenotype can be triggered through the expression of an enzyme that can modify sugar sinks called sucrose phosphate synthase according to by Kohorn et al. [40].

It has also been a well-adapted fact that WAKs can trigger many signal pathways leading to plants response to external stimuli such as pathogen attacks. In a study, treatment of protoplasts with pectin was observed to induce and repress hundreds of genes that are involved in biogenesis and responses of the plant cell wall, it was also observed that these genes were blocked in cells that had no *WAK2* [36, 40]. It was also proven that pectin can activate the expression of invertase enzyme but cannot do this in *wak2-1* null plants, meaning that *WAK1* and 2 can act as pectin receptors. It was also proven when the extracellular domain of *WAK1* was successfully fused into another cytoplasmic kinase EFR to create a novel chimeric receptor [82]. It is reasonable to conclude that many types of pectins have the ability to influence the role of WAKs in plant growth and disease response.

Numerous studies across rice, maize, Arabidopsis, wheat, and other cereal crops have consistently demonstrated that WAK receptor-like kinases contribute to enhanced resistance against fungal and bacterial pathogens, indicating that WAK-mediated immunity is broadly conserved among higher plants. WAK receptor-like kinases have been shown to enhance resistance against several diseases caused by fungi in rice [44], maize [81], Arabidopsis [45], cereals [46] and in many monocotyledonous plants [14, 44].

The WAK proteins identified as series of OsWAKs were reported by Delteil et al. [44] to trigger basal defense in rice plants against blast diseases through thickening of cell wall chitin, and this further strengthens the roles of WAK proteins in the enhancement of plant disease resistance. Receptor like protein kinases (RLKs) have been perceived to be highly conserved ligands than can enhance resistance in plants against some groups of pathogens, the *XA21* protein for example has been used to enhance resistance of banana plant against bacterial wilt pathogen called *Xanthomonas campestris pv. musacearum* [83]. In another instance, protein LRR-RLK EFR was observed to perceive bacterial elongation factor in arabidopsis, it was also observed to confer resistance in transgenic wheat against bacterial pathogen (*Pseudomonas syringe pv. oryzae*) causing halo blight disease [62].

It is now possible to modify endogenous receptor like protein kinases (RLPs) in crop plants and increase their resistance rate through genome editing where molecular differences between the susceptible alleles and the resistant ones are used to detect critical domains and amino acids for recognition [Table 2]. Morever, Marchal et al. [84], and Gomez De La Cruz [85] reveled that integrated domain (IDs) of NLRs (NLR-IDs) were found in many plant species and that about 3.5 to 10% of all the studied NRLs had additional integrated domains many of which were linked to plant immunity. It is generally believed that proteins that are similar to integrated domains can play vital roles in plant defense and a good knowledge of integrated domains could enhance the identification of their novel target effectors. Good analysis of NLR-IDs therefore has led to their identification for further targets and more understanding of plant host-pathogen relationships [84].

**Table 2:** Examples of plant species with characterized NLRs containing integrated domains (NLR-IDs)

NLR-ID Name	Plant Species	Integrated Domain(s)	Executor NLR (if known)	Pathogen Resistance
Pik-1	*Oryza sativa* (rice)	HMA-like, AAA	Pik-2	*Magnaporthe oryzae*
RGA5 (Pia-2)	*Oryza sativa*	HMA-like	RGA4 (Pia-1)	*M. oryzae*
Pii-2	*Oryza sativa*	NOI	Pii-1	*M. oryzae*
RRS1-R	*Arabidopsis thaliana*	WRKY	RPS4	*P. syringae*, *R. solanacearum*
RLM3_Col	*A. thaliana*	BRX (3x)	–	*L. maculans*, *B. cinerea*
Xa1/Xo1	*Oryza sativa*	ZnF_BED	–	*X. oryzae*
Yr5/Yr7/YrSP	*Triticum aestivum* (wheat)	ZnF_BED	–	*P. striiformis*
Rph15	*Hordeum vulgare* (barley)	ZnF_BED	–	*P. hordei*
RPP1-R1 to R5	*Glycine max* (soybean)	ULP1 protease	–	*P. pachyrhizi*
BnRPR1	*Brassica napus*	B3, TFSIIN	BnRPR2	–
OsRPR1	*Oryza sativa*	WRKY (x2)	OsRPR2	–
RGA2a	*Aegilops tauschii*	EXO70	RGA1e	–
RGH2	*Hordeum vulgare*	EXO70	RGH3	*B. graminis*
WRKY19	*A. thaliana*	PAH, WRKY (2x), MAPK	DSC1	*M. incognita*
Tsn1	*Triticum turgidum*	Pkinase	–	*P. nodorum*, *P. tritici-repentis*
YrU1	*Triticum urartu*	ANK, WRKY	–	*P. striiformis*
GmNLR-ID	*Glycine max*	WRKY	–	*R. solanacearum*
NLR-ID variants	*Solanum lycopersicum* (tomato)	Kinase, WRKY, DUF3542	–	*P. syringae*, *Fusarium spp.*
NLR-ID variants	*Capsicum annuum* (pepper)	Kinase, WRKY, TIR	–	*Xanthomonas spp.*
NLR-ID variants	*Zea mays* (maize)	Kinase, BED, WRKY	–	*Ustilago maydis*
NLR-ID variants	*Medicago truncatula*	WRKY, TIR, Kinase	–	*Aphanomyces euteiches*
NLR-ID variants	*Marchantia polymorpha*	CC-MAEPL motif	–	–

NLR-IDs = Nucleotide-binding, leucine-rich repeat receptor proteins, This table includes wild relatives, legumes, cereals, and basal land plants, showing the evolutionary breadth of NLR-ID integration. Many of these domains are recurrently integrated across lineages, suggesting convergent evolution for pathogen recognition

It is also interesting to know that a partial broad-spectrum resistant gene *Yr36* which encodes wheat kinase start1 (*WKS1*) protein. Wang et al. [86] previously detected it in *Triticum turgidum ssp. dicoccoides* (wild emmer wheat). As reported by Li et al. [87], *WKS1* confers a broad-spectrum resistance in this plant species against fungal stripe rust pathogen and later confirmed by Mourad et al. [88] and Kou and Wang [89]. Although *WKS1* is not a classical WAK receptor, its inclusion highlights the broader significance of kinase-mediated resistance pathways in crop protection. Similar to WAK-associated signaling, kinase-based resistance mechanisms provide valuable targets for the development of durable disease-resistant cultivars [39, 90].

Taken together, studies across diverse plant species consistently demonstrate that WAKs function as central regulators of cell wall integrity signaling. Although individual investigations have focused separately on development, stress adaptation, or disease resistance, these processes are linked through a common mechanism involving perception of cell wall-derived signals and activation of downstream kinase pathways. Thus, WAKs should not be considered solely as growth regulators or immune receptors, but rather as multifunctional signaling hubs that coordinate plant responses to both internal developmental cues and external environmental challenges.

## Conclusion

In conclusion, the pathogen attack, herbivory or wounding have been known to elicit the production of more oligogalacturonic acids (OGs) present in the plant cell wall, it has been established that OGs have specific receptor in the plant cell wall which is most likely to be WALKs. It is not clearly known how WAKs distinguish galacturonic acid from pectins, it is also not clearly understood how WAKs differentially bind to esterified and de-esterified pectins and how this resulted in their distribution in plant cells. Understanding of regulatory mechanism of different cell wall pectins on WAKs will reveal how they structurally bind to pectins, how they are distributed within the cell wall and roles they play in plant-pathogen relationships. Although it is known that the response of wall associated kinases (WAKs) to different kind of pectins in plants are controlled by genes which further affirm their roles in plant growth and pathogen/disease resistance. However, there are still dearth of knowledge on these genes despite their importance in biology, information is also not sufficient to prove the importance of WAK proteins in disease management.

The function of WAK’s linking the extracellular matrix with the cytoplasm and signaling the external conditions suggest a possibly mechanic-induction related with de-methylsterified pectins. *WAK1* for example is linked to cell wall pectin, as such, the stretching of de-methylesterified pectin tangles with calcium to form a structure popularly referred to as ‘egg-box’; a structure that can be identified by WAK’s. It is therefore possible that the changes in the biochemical composition of the wall itself are also important to address and well understood. It is also worth noting that oligogalacturonides production through pectin degradation mediated by polygalacturonases and pectate lyases, could be important in plant response to pathogens.

An important theme emerging from the literature reviewed here is that apparently diverse observations regarding growth regulation, pectin perception, pathogen recognition, and disease resistance can be unified through the concept of cell wall integrity sensing. WAKs serve as central components of this surveillance system, linking extracellular wall-derived signals with intracellular kinase-mediated responses. Therefore, many developmental and defensive functions previously considered independent are increasingly recognized as interconnected outcomes of WAK-mediated signaling.

A major emerging concept from recent studies is that WAKs function as cell wall integrity sensors that couple extracellular perception with intracellular signaling. Through their ability to recognize both native pectins and pathogen-induced oligogalacturonides, WAKs integrate developmental and defense-related signals into common kinase-dependent signaling networks. Activation of MAPKs, ROS-mediated responses, defense-associated transcription factors, and cell wall remodeling genes collectively explains how WAKs regulate apparently diverse biological processes through a unified mechanism.

The knowledge about all the signaling pathway that is triggered in biotic and abiotic stress processes is barely being deciphered by the multiple work that has been done in recent years, including the WAK’s. However, progress in identification and understanding of WAKs, DAMPs-TI and DAMP-PRR pairs and relationships as explained in this writeup will enhance breeding of crops that are resistant to many pathogens/diseases.

## Data Availability

No datasets were generated or analysed during the current study.
